# Glucose Injections at Birth, Warmth and Placing at a Nurse Sow Improve the Growth of IUGR Piglets

**DOI:** 10.3390/ani9080519

**Published:** 2019-08-02

**Authors:** Maiken N. Engelsmann, Christian F. Hansen, Marlene N. Nielsen, Anders R. Kristensen, Charlotte Amdi

**Affiliations:** 1 Department of Veterinary and Animal Sciences, Faculty of Health and Medical Sciences, University of Copenhagen, DK-1870 Frederiksberg C, Denmark; 2 Pig Research Centre, Danish Agriculture and Food Council, Axeltorv 3, DK-1609 Copenhagen V, Denmark

**Keywords:** colostrum supplementation, glucose injection, Intrauterine Growth Restriction, piglets, rectal temperature

## Abstract

**Simple Summary:**

Selection for hyperprolific sows has increased litter sizes and increased the number of small piglets per litter that require more management. Some of these small piglets have been exposed to intrauterine growth restriction, making them even more susceptible to a lower vitality and a higher mortality during the first few days. Administering an energy bolus at birth—such as glucose together with exposure to extra warmth—could be one way of increasing the growth and vitality of small, less viable piglets, ensuring piglet gain and survival. In addition, the results are relevant when in a relatively non-competitive environment—for example, placing them at a nurse sow suitable for rearing small piglets.

**Abstract:**

Intrauterine growth-restricted piglets (IUGR) have a lower rectal temperature, whole-blood glucose, and lower glycogen storages at birth than normal piglets, giving them less energy to maintain body temperature and compete at the udder. The present paper investigated the effects of giving an energy supplementation three times after birth on rectal temperature, glucose levels, and growth until weaning in an on-farm trial. Eighty-eight newborn piglets were classified as IUGR (based on head morphology), placed under a heating lamp for one hour and allocated to one of four treatments—warmed water (WATER), glucose injection (GLUC), colostrum bolus (COLOS; porcine colostrum), and colostrum bolus and glucose injection (GLUC + COLOS)—before being placed at a nursing sow. Weight differences were found at day 21, with GLUC and GLUC + COLOS groups being the heaviest. Piglets in GLUC + COLOS had higher glucose levels at *t* = 3, 6, and 9 h compared to the other treatments (*p* = 0.027), but from *t* = 24 h and onwards, no difference was observed. For rectal temperature, no differences were observed. Collectively, these findings suggest that glucose injections at birth (i.e., as an energy source), one hour’s exposure to warmth and the placement of piglets with a nurse sow to reduce competition, enhance the growth of IUGR piglets.

## 1. Introduction

Genetic selection towards highly prolific sows has resulted in increased litter sizes, leading to a significantly lower mean birth weight and an increased percentage of small piglets born [1]. This increase in litter size has caused intrauterine crowding and a higher demand for oxygen and nutrients during gestation [2]. Consequently, approximately 30% of neonatal pigs in Denmark suffer from various degrees of intrauterine growth restriction (IUGR) [3,4]. Intrauterine growth restriction is defined as the impaired growth and development of the foetus and/or its organs during gestation [5], and because of the brain-sparing effect [6], IUGR piglets can be identified by their unique head shape [3,7].

At birth, an initial decrease in rectal temperature is observed in IUGR piglets, and it can take up to 48 h after birth until the normal body temperature of 39 °C is re-established [8]. To re-establish and maintain the body temperature, newborn piglets rely on glycogen deposits, colostrum intake, and shivering thermogenesis [9]. Intrauterine growth-restricted piglets have a larger surface area to volume ratio than normal piglets and, therefore, exhibit greater heat loss, making them more susceptible to hypothermia [9]. In addition, IUGR piglets exhibit lower hepatic glycogen deposits [4], which, in normal piglets, are depleted approximately 16 h after birth [10]. Therefore, in the first few hours, it is critical for the IUGR piglets to start suckling in order to maintain their body temperature and receive the required energy to survive. Additionally, IUGR piglets have lower rectal temperatures at birth than normal piglets—36.2 vs. 37.5 °C respectively [11]—and surviving piglets at day seven post-partum were found to have a rectal temperature at birth of 37.7 °C compared to 36.5 °C in piglets that died [12]. In addition, it has been estimated that IUGR piglets do not ingest sufficient amounts of colostrum [4], compared to the 200 g colostrum recommended per piglet over the first 24 h to ensure survival during the neonatal phase [13]. Administering both heat and a bolus of colostrum increased rectal temperature by 1 °C one hour after birth in IUGR piglets, however, the effect disappeared after four hours [14], suggesting that an energy supplementation needs to be given frequently.

Therefore, the objective of the present study was to investigate the effect of administering IUGR piglets with an energy supplementation three times during the first 24 h after birth, on whole-blood glucose, body weight (BW) gain, and rectal temperatures from birth to day 21 in an on-farm trial. Piglets were placed under a heating lamp for one hour and placed at the same nursing sow suitable for rearing small piglets, to eliminate competition and variability within treatment. We hypothesised that a bolus of porcine colostrum or glucose injection, or both, at birth would have a positive effect on the BW gain, whole-blood glucose levels and rectal temperatures from birth to day 21 in IUGR affected piglets.

## 2. Materials and Methods

### 2.1. Ethical Approval

The experiment was carried out with respect to ethical procedures for animal experimentation and with approval from the Danish Animal Experimentation Inspectorate, license number 2016-15-0201-01018.

### 2.2. Experimental Design

A total of 88 IUGR piglets were included in the experiment from 37 sows with a parity between 1 and 7. The piglets were visually graded and classified as IUGR piglets based on the modified characteristics from Hales et al. [3] and Chevaux et al. [7]. The piglets were given a visual score, determining the IUGR piglets by a (1) steep dolphin-like forehead, (2) bulging eyes, (3) hair with no direction of growth. A piglet was defined as IUGR if it had a steep dolphin-like forehead and one other of the characteristics were present; if none of the characteristics were present, the piglets were considered normal and not used in the experiment. A visual score based on the piglets’ head shape was chosen, as this has previously been found to be an easier identification method for the farmer than birth weight (see discussion in [14,15]).

At time = −1 h, newborn (approximately between 1–12 h of age) piglets used in the experiment were ear tagged, their sex was recorded, their whole-blood glucose was measured using a glucose monitor (Accu-Chek Aviva Nano, Roche, Basel, Switzerland), a drop of blood was obtained by puncturing the ear vein of each with a syringe (23G × 1-Nr.16, Kruuse, Langeskov, Denmark), rectal temperatures were measured (Apotekets digitaltermometer standard, Apotekets, Hørsholm, Denmark), BW was recorded (UWE, Bjerringbro Vægte ApS, Bjerringbro, Denmark) and the birth sow’s parity and litter size were noted.

The experimental IUGR piglets were allocated to one of the four treatments (*n* = 22) and blocked according to their rectal temperature, measured at *t* = −1 (ranging from 33 to 39 °C). The four treatments were (1) Water (WATER; 20 mL 35 °C warmed water) tube-fed (Unomedical feeding tube, Hatting, Horsens, Denmark); (2) Glucose (GLUC; Glucose Baxter Viaflo, DK Baxter A/S, Allerød, Denmark) (50 mg/mL) injected subcutaneously (4 × 1.5 mL, two in the groin area and two in the neck); (3) Pooled porcine colostrum (COLOS; 20 mL warmed to 35 °C) tube-fed, previously obtained from other sows around farrowing in the same piggery, and (4) both glucose injected subcutaneously and pooled porcine colostrum tube-fed as described above (GLUC + COLOS). After allocation to one of the four treatments, the piglets were placed in a boarded-off creep area for one hour with a heating lamp (from *t =* −1 to 0). Treatments were administered three times, at *t =* 0, 3, and 6 h. Rectal temperatures, whole-blood glucose levels, and BWs were measured before each treatment, and at 9, 12, 24, 36, 48 h and at days 7, 14 and 21. The piglets were placed at a nurse sow, with which they stayed for the duration of the trial after the first administration. The nurse sows were selected on the basis of maternal quality; i.e., the number of weaned piglets in the last parity, if they had 14 functional teats and if they had just farrowed (day 0 post-partum). At days 7, 14, and 21 post-partum, glucose levels, rectal temperatures, and BWs were measured and blood samples obtained. The blood samples were collected by holding the piglets in dorsal recumbence, and 6 mL of blood was drawn from the jugular vein using 22-gauge needles into Vacutainer tubes containing EDTA (BD Vacutainer, Franklin Lakes, NJ, USA) and placed on ice until centrifugation. The samples were centrifuged at room temperature for 15 min at 1200× *g* (CM-6MT, Elmi, Riga, Latvia), after which, the plasma was transferred to Eppendorf tubes (Sarstedt, Nümbrecht, Germany) and immediately frozen at −20 °C for later analysis of the plasma IGF-1. The plasma IGF-1 concentrations were measured using a porcine IGF-1 ELISA kit (Nordic Biosite, Tæby, Sweden) according to the manufacturer’s recommendations. Samples were performed in duplicate and the intra-assay variation was 3.3%. The inter-assay variation was 12.6%.

The experimental period lasted from day 0 post-partum until day 21 post-partum, when the piglets were sampled a final time and the experiment ended. One nurse sow was made per week where all four treatments were present at the same sow, adding up to twelve piglets in total (three from each treatment). In order to put 14 piglets to the sow, two treatments would have one extra piglet one week and the two other treatments would have one extra piglet represented at the nurse sow the following week, alternating each week. Only experimental piglets were given to the sow and no replacements were made if experimental piglets died.

### 2.3. Housing and Animals

The study was conducted in a commercial Danish piggery (Rødvig Stevns, Denmark) with 1200 Danish Landrace × Danish Yorkshire sows. The sows were artificially inseminated with Duroc semen (Hatting KS, Horsens, Denmark). The farrowing unit consisted of nine sections, where all farrowing pens were 3.9 m^2^, with cast iron slats and an insulated concrete floor. The individual farrowing crates each had a creep area for the piglets of approximately 0.53 m^2^, with a cover and floor heating and radiation from an infrared 150 W heating lamp providing a temperature range of 27–28 °C in the creep area. The heating lamp was turned on when the first piglet was observed and turned off at day 5 post-partum. The ambient temperature in the farrowing unit was 22 °C while the sows were farrowing, which was controlled via negative pressure ventilation with an exhaust chimney in the middle of each section, and the room temperature was decreased by 1 °C each week.

### 2.4. Feeding Practice and Diets

From days 4–5 pre-partum to day 6 post-partum, all sows were fed the same commercially formulated diet based on barley, wheat, soy, and sugar beet pulp as the main ingredients, with an energy level of 13.02 ME/kg and 107 g/kg digestible crude protein. They were fed this diet two times a day as liquid feed, until all sows in the sections had finished farrowing, after which, the sows were fed three times daily. Besides this, the sows were provided with a handful of straw each day until farrowing. At day 6 post-partum, the lactating sows were switched to a diet based on the same main ingredients, but with energy levels of 13.40 ME/kg and 131 g/kg digestible crude protein. The feed was provided three times a day as liquid feed. After each feeding, all sows were observed for major feed refusals and individual feed curves adjusted accordingly. Sows with feed residues were then observed for illness. From day 5 and onwards to weaning, piglets were offered a weaner diet on the floor (PrimeConc Midi Müsli U, DLG, Copenhagen, Denmark) consisting of 29.5% crude protein, 6.1% crude fat, 2.2% fibre and 8.1% crude ashes.

### 2.5. Management and Monitoring in the Farrowing Unit

The farrowing sows were monitored during the daytime (07:00–15:00 h) and then again at 20:00 h, where obstetric aid was provided if no piglets had been born within the last hour or if the sows previously had a history of many stillborn piglets. Each morning at 08:00 h, all newborn piglets with dried umbilical cords were treated with Amoxicillin (Clamoxyl^®^, Orion Pharma Animal health, Copenhagen, Denmark) and the umbilical cord was shortened and sprayed with a disinfection product (0-infektion, Aeropak A/S, Hedensted, Denmark). The piglets were provided with a milk replacer (Elitemilk Pigi, Vilofarm, Hobro, Denmark) during the following 5 to 7 days and a board with a hole closing off the creep area for warmth, but which also allowed the piglets to enter or leave the creep area. All experimental IUGR piglets were managed in accordance with the general routines on the farm; all piglets were tail docked and given an iron injection (Solofer vet^®^, Pharmacosmos, Holbæk, Denmark) and Baycox injection against coccidiosis (Baycox^®^, Bayer Animal health division, Copenhagen, Denmark) at days 3 to 4 post-partum. The castration of male piglets was performed surgically with post-operative analgesia (Flunixin vet^®^, Scanvet Animal Health A/S, Fredensborg, Denmark). Stock personnel inspected the health of the animals each day, and usual management practices for treatments and vaccinations were followed. If an IUGR piglet died, the date and ear tag was registered. Any sows with mastitis-metritis-aga-lactia syndrome (MMA) were treated with either Amoxicillin (Curamox^®^, Boehringer Ingelhem, Copenhagen, Denmark) or with trimetoprim and sulfonamid (Tribissen, MSD, Ballerup, Denmark).

### 2.6. Statistical Analyses

All statistical analyses were performed using the statistical software SAS (GLM procedure of SAS; SAS Inst.Inc., Cary, NC, USA), with the piglet as the experimental unit and analyzed by 3-way repeated measures ANOVA (time × glucose × colostrum). Primary test parameters were: piglet BW, whole-blood glucose levels, and rectal temperature. In the model, sex was included, the nurse sow was included as a random factor, and the piglet ID as a repeated measurement and body weight at *t*= −1 were included as covariates. All possible interactions were tested, and if *p* > 0.05 then it was eliminated from the final model. The results are presented as least square means and standard errors of means (SEM). The data were divided into measurements made during the first 48 h, and measurements made at *t =* 48 h and during the subsequent 21 days. This gave two final models, one over hours and one over days for each measurement (whole-blood glucose, rectal temperature, and BW). Statistical significance was accepted at *p* < 0.05 and *p* < 0.10 was considered a tendency.

## 3. Results

The study included 88 IUGR piglets, of which 34 died before day 21 (38.6%). In GLUC + COLOS, five of the 22 piglets in the group died, compared to 10 in WATER and GLUC, and nine in COLOS. Fifteen of the 34 dead piglets died during the first week (44.1%). The IUGR piglets in this experiment weighed 0.74 ± 0.02 kg at birth, had glucose values of 2.8 ± 0.2 mmol/L, and rectal temperatures of 36.6 ± 0.1 °C. The means of the four groups can be seen in Table 1. Of the 88 IUGR piglets, 46 were males, 41 were females and one was unaccounted for (WATER: 14 male, 8 female; GLUC: 8 male, 14 female; COLOS: 12 male, 10 female; GLUC + COLOS: 12 male, 9 female). At the end of the experiment, when the piglets were 21 days old, they weighed 4.8 ± 0.1 kg, had whole-blood glucose values of 6.0 ± 0.2 mmol/L, and a rectal temperature of 39.1 ± 0.1 °C.

### 3.1. Body Weight (BW)

#### 3.1.1. First 48 h

There was a colostrum × glucose interaction (*p* < 0.001, F_1,712_ = 14.36) on BW over the first 48 h. Piglets in the WATER group weighed on average 0.80 ± 0.01 kg; in the GLUC group, 0.79 ± 0.01 kg; in the COLOS group, 0.80 ± 0.01 kg; and in the GLUC + COLOS group, 0.82 ± 0.01 kg. The GLUC + COLOS group were on average heavier than all the other groups (*p* < 0.01). The COLOS group was heavier than the GLUC group (*p* = 0.038). The WATER group was heavier than the GLUC group (*p* < 0.01), but with no difference compared to the COLOS group (*p* = 0.535).

#### 3.1.2. Effect of Days

**Figure 1:**
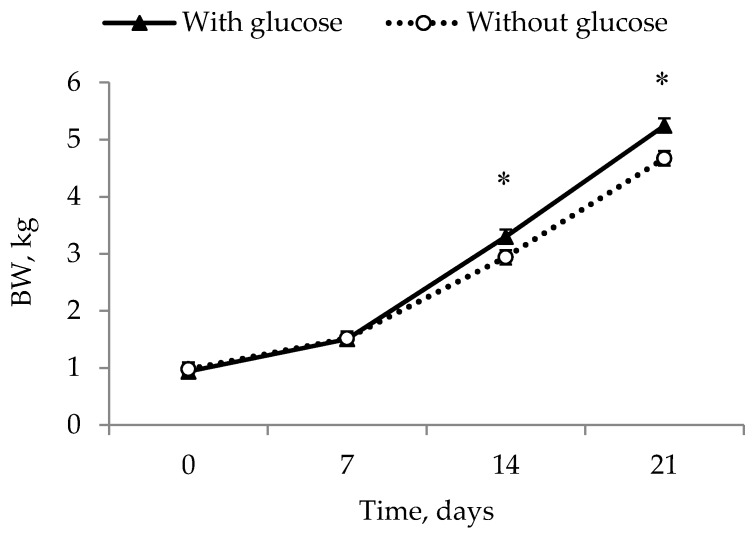
Body weight (BW) values at days 2, 7, 14 and 21 for intrauterine growth-restricted piglets (IUGR) piglets given glucose (▲) or without glucose (○). * Represents a significant difference between treatments (*p* < 0.05). The values presented are least squares means ± SEM.

There was an effect of day × glucose interaction (*p* < 0.03, F_3,234_ = 3.05) on BW over the 21 days of lactation (Figure 1), with higher levels in the piglets having received glucose. There was an effect of day with increasing weights throughout the trial (0.96, 1.52, 3.12 and 4.96 kg SEM 0.09; *p* < 0.001, F_3,234_ = 435.49), an effect of glucose (with; 2.75 kg; without 2.53 kg SEM 0.06; *p* = 0.013, F_1,234_ = 6.34), and no effect of colostrum (*p* = 0.932, F_1,234_ = 0.01). Piglets given glucose injections ended up with the highest BW at the end of the experiment; GLUC, 5.25 ± 0.19 kg and GLUC + COLOS, 5.18 ± 0.17 kg, compared to WATER, 4.69 ± 0.21 kg, and COLOS, 4.34 ± 0.21 kg.

### 3.2. Whole-Blood Glucose

#### 3.2.1. First 48 h

**Figure 2:**
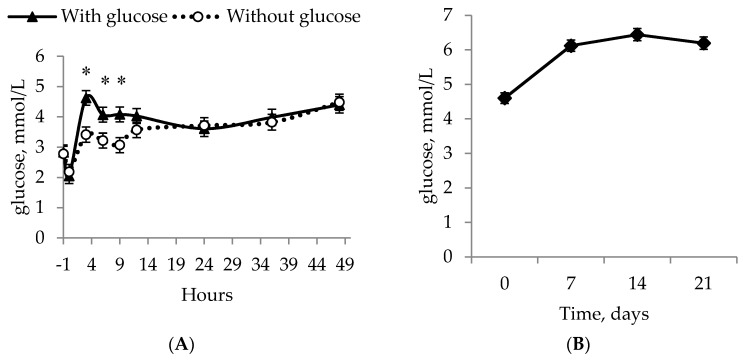
Whole-blood glucose values over the first 48 h (**A**) for IUGR piglets given glucose (▲) or without glucose (○) and the overall glucose level at days 2, 7, 14 and 21 (**B**). * Represents a significant difference between treatments (*p* < 0.05). The values presented are least squares means ± SEM.

There was an effect of hours × glucose interaction (*p* = 0.026, F_8,727_ = 2.20; Figure 2A) on whole-blood glucose over the first 48 h and a colostrum × glucose interaction (*p* = 0.001, F_1,727_ = 14.16). There was no difference between WATER, GLUC and COLOS with levels of 3.5 mmol/L, 3.4 mmol/L, and 3.3 mmol/L respectively (SEM 0.12 mmol/L; *p* > 0.05), but GLUC + COLOS had higher values than the other groups (4.1 mmol/L). All of the piglets experienced the same drop in whole-blood glucose from *t =* −1 h to 0 h (Figure 2A), where they were confined in the creep area with a heat source.

#### 3.2.2. Effect of Days

The blood glucose concentration over time is shown in Figure 2B. Time differences for glucose concentrations were seen between week one and the following three weeks (*p* < 0.001, F_3,236_ = 27.31). There was no effect of glucose (*p* = 0.721, F_1,236_ = 0.13), but a tendency for an effect of colostrum (with colostrum, 6.0 mmol/L and without, 5.7 mmol/L SEM 0.12 mmol/L, *p* = 0.083, F_1,236_ = 3.04).

### 3.3. Rectal Temperature

#### 3.3.1. First 48 h

**Figure 3:**
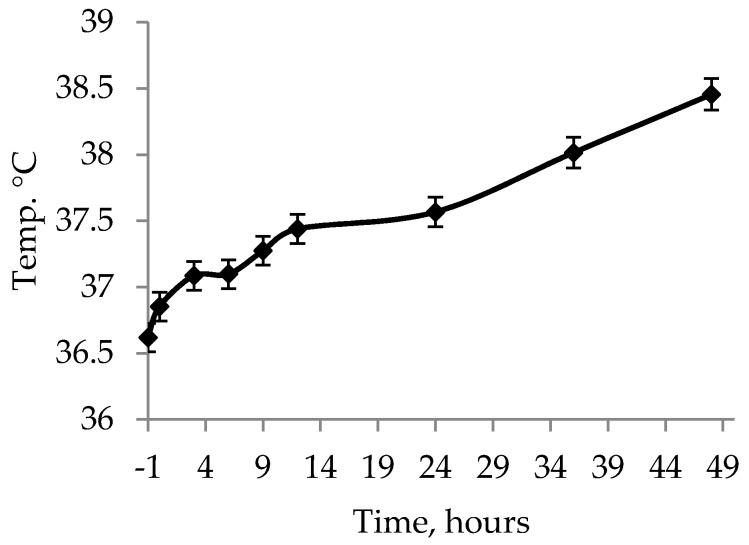
Overall rectal temperature values over the first 48 h for IUGR piglets. The values presented are least squares means ± SEM.

There was an effect of hours on rectal temperature over the first 48 h with increasing values (*p* < 0.001, F_8,743_ = 25.27; Figure 3). There was a tendency for an effect of colostrum (with colostrum, 37.5 °C; without colostrum, 37.3 °C SEM 0.05; *p* = 0.068, F_1,743_ = 3.35), but no effect of glucose (*p* = 0.658, F_1,743_ = 0.20).

#### 3.3.2. Effect of Days

There was an effect of days on rectal temperature with increasing values over days; 38.5, 38.6, 39.2 and 39.1 °C (SEM ± 0.09 °C; *p* < 0.001, F_3,235_ = 13.16). There was no effect of colostrum (*p* = 0.209, F_1,235_ = 1.59) or glucose (*p* = 0.07, F_1,235_ = 3.55).

### 3.4. Plasma IGF-1

There was an effect of days on plasma IGF-1 concentrations with higher concentrations in week 2 than weeks 3 and 4 (78.7, 56.3, 48.3 ng/mL SEM; 3.74 ng/mL; *p* < 0.001, F_2,91_ = 18.55) respectively. There was a tendency for an effect of glucose (with 57.1 ng/mL and without 65.1 ng/mL; SEM 3.04 ng/mL; *p* = 0.07, F_1,91_ = 3.36), but no effect of colostrum (*p* = 0.880, F_1,91_ = 0.02) on plasma IGF-1.

## 4. Discussion

This experiment was designed to study the effect of giving an energy supplement—such as glucose or colostrum—at three time points during the first 24 h of life on the subsequent growth performance of IUGR piglets. Intrauterine growth-restricted piglets have a much higher mortality rate (up to 60%) than normal piglets within the first 48 h of life [3] and management strategies could be one way to improve survival. In the present experiment, 38% died before day 21. This suggests that management interventions can prevent a substantial amount of IUGR piglets from dying. It is, however, imperative that interventions are easy to perform and not too time consuming for them to be relevant for the farmer. The results demonstrated that giving glucose injections or a colostrum bolus in combination with heat and placement at a nurse sow did, overall, result in a small increase in whole-blood glucose concentration, but with no differences found in rectal temperatures during the first 24 h. At weaning, however, the IUGR piglets receiving glucose injections were heavier than the other treatments. All of the piglets were exposed to one hour of heat to increase body temperature—as this is vital for survival [12]—and were placed at a nurse sow to eliminate competition from larger littermates. This experimental design does, however, mean that it is not completely possible to elucidate the effects of these different factors. In addition, a true control to the glucose injection would have been a saline injection. However, as the aim was to simulate an on-farm situation, this was decided upon as being too invasive as a control group. This does, however, limit the interpretation of this study. What can be concluded though, is that energy, warmth and the removal of competition are valuable management tools in preventing IUGR piglets from dying and in giving them the best possible opportunities for growth.

In the present experiment, 20 mL of porcine colostrum was administered three times. The allocation of 20 mL per treatment was based on a recommendation from Amdi and Hales [16], who found that the maximum capacity of a 1 kg piglet’s stomach was 34 mL when inflated to the absolute maximum. Other studies that have tried to enhance the colostrum intake of piglets to increase their growth have primarily focused on supplementation with porcine colostrum [14,17,18]. Body weight gain was found to increase when low birth weight piglets were given one oral supplementation of 15 mL of porcine colostrum [17]. This was not the case in the present study, where COLOS did not result in a higher BW compared to the WATER group. Piglets dehydrate very quickly and it can be speculated that the WATER group in fact became more hydrated [14] and that this is part of the reason that no difference was found compared to the COLOS group. Further, it can be speculated that the colostrum treatment in the COLOS group reduced suckling motivation or that using colostrum supplementation as a mean of increasing the BW of IUGR piglets is simply not enough, and that a more direct energy source such as a glucose injection is more efficient. Both GLUC and GLUC + COLOS ended up with a higher BW at day 21, supporting this hypothesis. This difference shows that IUGR piglets given a glucose injection in some way had extra energy, which is perhaps necessary to compete at the udder or to use towards growth. Impaired growth in IUGR piglets has been coupled to a functional defect in the Insulin-like growth factor-system (IGF-system) [19]. Intrauterine growth-restricted piglets do not exhibit the same levels of IGF-1 in the plasma, in skeletal muscle, or in the mucosa of the gastrointestinal tract [20], nor do they exhibit the increased pattern of IGF-1 in the pre- and post-natal transition period [21] compared to their normal littermates. The extra energy from the glucose injections has potentially slowed down the utilization of glycogen reserves.

All of the piglets managed to increase their glucose levels and followed the same pattern during the experiment, with the fastest increase happening for IUGR piglets in the GLUC + COLOS group. The greater glucose levels GLUC + COLOS experienced were 3 h after each allocation (*t =* 3, 6, 9) compared to the other treatments; however, after *t =* 24 and onwards, no difference was observed. It was shown that IUGR piglets have longer and thinner intestines [22], which may impair their utilization of glucose. In addition, IUGR piglets have fewer intestinal microvilli and a reduced height of intestinal villi [23], suggesting that IUGR piglets perhaps cannot absorb glucose or other nutrients at the same rate as their normal littermates. This could be why piglets in GLUC + COLOS had higher glucose levels, as these piglets may quickly use the energy from the glucose injection because it circumvents the absorption from the gut, thereby making sure glucose levels remain elevated. This was followed up by the natural consumption of colostrum. Another possible reason for elevated blood glucose could be that glucose injections increased the blood glucose concentration faster and thus the IUGR piglets in GLUC + COLOS did not experience hypoglycemia, defined as a glucose concentration at or below 2.8 mmol/L [24] for as long. Even though GLUC + COLOS increased the piglets’ whole-blood glucose levels at some time points, all treatment groups ended with no difference between them and an average whole-blood glucose level of 6.0 ± 0.2 mmol/L.

Besides the treatments and placing at a nurse sow, one hour of heat was administered to all piglets by enclosing the piglets in the creep area with a heating lamp to stabilize their rectal temperature. This was important, as piglets that die have been found to have lower rectal temperature than surviving littermates [12]. The one hour of heat cannot compensate for the chilling caused by the ambient temperature outside of the womb, however, the one hour of heat managed to ensure that the rectal temperatures stabilized or increased for the piglets between *t =* −1 and *t =* 0. Due to it being an on-farm trial it was, however, not possible to collect the piglets at birth but, instead, before farm staff moved the piglets around.

Placement at a nurse sow was done to limit variation (all treatments were at the same sow and there was no competition from larger littermates). The combination of supplementation with extra farrowing care, such as split nursing on the difference in mortality of low-birth-weight-piglets, was investigated by Holyoake et al. [25]. They provided low-birth-weight-piglets with between 10 and 15 mL of colostrum either by stomach-tube or bottle, after which split-nursing was performed to litters with 12 piglets, resulting in a lower mortality of low-birth-weight-piglets [25]. Dewey et al. [26] also observed the difference in a “maximal care” group compared to what was normal procedure at the farm, “standard care”. In the “maximal care” group, the chilled piglets were either orally given 12 to 20 mL of porcine colostrum or 3 mL of 10% solution of glucose, and split-nursing was performed for 1 h in litters with more than 12 piglets. Piglets in the “maximal care” treatment obtained an increase in piglets’ BW at day 16 (BW 4.8 kg standard care vs. 4.9 kg in maximal care) and a tendency towards reduced mortality [26], in line with the findings from the present study. In the present study, the nurse sow could not be eliminated as a factor in any of the statistical models, proving the importance of the sow. Su et al. [27] also claimed, in a pilot study, that nursing sows had a minor positive effect on piglet survival rates during the first 5 days post-partum.

The genetic selection towards highly prolific sows has increased the number of small piglets in a litter, through the inverse correlation between piglet birth weight and litter size [28]. In addition, litter size negatively affects the survival of the piglets, with an increased number of stillborns per litter and a higher piglet mortality [1]. In the present experiment, a large litter size affected rectal temperatures and BWs during the first 48 h. Also, sex has previously been reported to affect rectal temperatures. Baxter et al. [29] found lower rectal temperatures in males, suggesting that male piglets could have impaired thermoregulation compared to females, but more research is needed to confirm this. Vaillancourt and Tubbs [30] suggested that the increased risk of dying in males could be due to testosterone, which increased the energy-expensive processes of degrading and producing protein, thereby reducing the energy available to regain body temperature and acquire colostrum. This could indicate a difference between sex when it comes to responses to energy supplementation, or possibly udder seeking behavior, as Baxter et al. [29] found a tendency for males to be crushed more often, and Bate et al. [31] found, in a study of teat-seeking ability, that males tend to suckle later than females. A significant effect of sex was, however, not observed in the present experiment.

## 5. Conclusions

In conclusion, the glucose groups ended up with the highest BW gain at weaning, suggesting that the administering of glucose injections as an energy source, one hour’s exposure to warmth, the placement of piglets with a nurse sow to reduce competition, and access to a milk replacer for 5 to 7 days post-partum, all improve the growth of IUGR piglets. In addition, a small increase in whole-blood glucose concentration was found for the GLUC + COLOS group, but no differences in rectal temperatures were observed. Energy, warmth and the removal of competition are valuable management tools in preventing IUGR piglets from dying and giving them the best possibility for growth.

## Figures and Tables

**Table 1:** The characteristics of the piglets at time −1 (beginning of the trial) in the four groups; warmed water (WATER), glucose injection (GLUC), colostrum bolus (COLOS), and colostrum bolus and glucose injection (GLUC + COLOS).

Treatment	WATER	GLUC	COLOS	GLUC + COLOS	SEM	*p-*Values
*n*	*22*	*22*	*22*	*22*		
Body weight, kg ^1^	0.74	0.78	0.74	0.76	0.03	0.660
Glucose, mmol/L ^2^	2.8	2.7	2.7	3.0	0.4	0.468
Rectal temperature, °C ^3^	36.7	36.6	36.6	36.6	0.3	0.795

^1^ BW, F_1,84_ = 0.19, ^2^ Glucose F_1,83_ = 0.53, ^3^ Rectal temperature, F_1,84_ = 0.07.
